# Performance Monitoring in Medication-Naïve Children with Tourette Syndrome

**DOI:** 10.3389/fnins.2016.00050

**Published:** 2016-03-01

**Authors:** Heike Eichele, Tom Eichele, Ingvar Bjelland, Marie F. Høvik, Lin Sørensen, Heidi van Wageningen, Marius Kalsås Worren, Kenneth Hugdahl, Kerstin J. Plessen

**Affiliations:** ^1^Department of Biological and Medical Psychology, University of BergenBergen, Norway; ^2^K.G. Jebsen Centre for Research on Neuropsychiatric Disorders, University of BergenBergen, Norway; ^3^Section for Neurophysiology, Department of Neurology, Haukeland University HospitalBergen, Norway; ^4^Department of Psychiatry, Haukeland University HospitalBergen, Norway; ^5^Department of Clinical Medicine, Faculty of Medicine and Dentistry, University of BergenBergen, Norway; ^6^Child and Adolescent Mental Health Center, Mental Health Services Capital RegionCopenhagen, Denmark; ^7^Department of Clinical Medicine, Faculty of Health and Medical Sciences, University of CopenhagenCopenhagen, Denmark

**Keywords:** Tourette syndrome, ADHD, children, P3, event-related potentials, performance monitoring

## Abstract

**Background:** Tourette syndrome (TS) is a childhood-onset neurodevelopmental disorder and its impact on cognitive development needs further study. Evidence from neuropsychological, neuroimaging and electrophysiological studies suggests that the decline in tic severity and the ability to suppress tics relate to the development of self-regulatory functions in late childhood and adolescence. Hence, tasks measuring performance monitoring might provide insight into the regulation of tics in children with TS.

**Method:** Twenty-five children with TS, including 14 with comorbid Attention-deficit/ hyperactivity disorder (ADHD), 39 children with ADHD and 35 typically developing children aged 8–12 years were tested with a modified Eriksen-Flanker task during a 34-channel electroencephalography (EEG) recording. Task performance, as well as stimulus-locked and response-locked event-related potentials (ERP) were analyzed and compared across groups.

**Results:** Participants did not differ in their behavioral performance. Children with TS showed higher amplitudes of an early P3 component of the stimulus-locked ERPs in ensemble averages and in separate trial outcomes, suggesting heightened orienting and/or attention during stimulus evaluation. In response-locked averages, children with TS had a slightly higher positive complex before the motor response, likely also reflecting a late P3. Groups did not differ in post-response components, particularly in the error-related negativity (ERN) and error-related positivity (Pe).

**Conclusions:** These findings suggest that children with TS may employ additional attentional resources as a compensatory mechanism to maintain equal behavioral performance.

## Introduction

Tourette Syndrome (TS) is a childhood onset neuropsychiatric disorder with multiple motor tics and at least one vocal tic for more than 1 year (American Psychiatric Association, 1994). Tics are often described as semi-voluntary, because children with TS can suppress their tics for a certain amount of time at the cost of increasing discomfort for the patient (Spessot et al., 2004). However, tic suppression is tiring and effortful, and may contribute to an increased feeling of “premonitory urge,” which is an unpleasant bodily sensation preceding a tic and relieved by tic expression (Leckman, 2002). This reduction of unpleasant bodily sensation may contribute to a negative reinforcement of tic performance habit (Plessen, 2013).

Tic symptoms often attenuate in adolescence and about 40% of children are tic-free at the age of 18 (Leckman et al., 1998; Burd et al., 2001; Bloch and Leckman, 2009). This typical course of symptoms suggests that individuals with TS constantly, and often unconsciously, aim to suppress emerging tics to improve their psychosocial function (Eichele and Plessen, 2013). This process coincides with the development of self-regulatory control during childhood and adolescence (Davidson et al., 2006; Tau and Peterson, 2010) and maturation of the frontal cortex (Gogtay et al., 2004).

The ability to dynamically adapt the behavior to situational demands is a crucial part of adequate daily functioning (Ullsperger, 2006; Ullsperger et al., 2014). This requires a set of processing functions that localize to a broad network of brain areas encompassing frontal cortices, basal ganglia and thalamic nuclei, the cortico-striato-thalamo-cortical (CSTC) circuits. Activity in this network is elicited during performance monitoring and can be tested with the Eriksen-Flanker task (Eriksen and Eriksen, 1974).

Attention networks contribute to the perception of environmental cues that is essential for regulating behavior (Posner et al., 2014), and thus underlie the capacity of self-regulation (Rothbart et al., 2011). Different tasks of performance monitoring have been widely used to study this form of control (Fan et al., 2002). Recent work indicates that inhibitory control networks involving CSTC circuits are engaged during conflict trials to prevent attentional capture and interference (Tau and Peterson, 2010). Finally, imaging studies of individuals with TS implicate that inhibitory cognitive control processes might be altered (Worbe et al., 2015).

Due to the assumption that persons with TS show impairment of the CSTC circuits and the overlap of these networks with those involved in performance monitoring, the latter may also show impaired function. However, multiple studies report comparable, or even superior abilities of motor and cognitive control in children with TS compared with controls (Ozonoff and Jensen, 1999; Serrien et al., 2005; Mueller et al., 2006; Jackson et al., 2007, 2011; Eichele et al., 2010a). It is therefore of interest to investigate possible adaptive effects in this network. Many persons with TS are co-diagnosed with at least one further psychiatric disorder, with attention-deficit/hyperactivity disorder (ADHD) being the most common comorbid condition with 50–60% of all Tourette syndrome patients (Robertson, 2012; Hirschtritt et al., 2015). The reasons for the high co-occurrence have been widely discussed in the last decades but exact mechanisms still remain unclear. Evidence suggests that deficits in the basal portions of CSTC circuits represent shared neurobiological substrates for both disorders (Vloet et al., 2006; Sobel et al., 2010). Studies comparing children with TS with and without comorbid ADHD implied that children with comorbid ADHD showed impaired performance in tasks demanding cognitive control (Roessner et al., 2007; Greimel et al., 2008, 2011; Sukhodolsky et al., 2010). This is in line with findings suggesting altered behavioral and electrophysiological measures of performance monitoring tasks in persons with ADHD (Barry et al., 2003; Liotti et al., 2005; Johnstone and Galletta, 2013; Johnstone et al., 2013).

Different trial types modulate the sequence of stimulus- and response-locked event-related potentials (ERP) in the electroencephalogram (EEG) and outcomes indicate modulations of interference/conflict and control. The stimulus-locked N2 reflects early stages of conflict/mismatch detection (Folstein and Van Petten, 2008; Larson et al., 2014). This component is also reduced in children with ADHD (Albrecht et al., 2008). We decided to focus on the subsequent P3 that is thought to reflect a neural representation of a sensory process where the incoming stimulus is compared to the mental representation of the previous stimuli and the stimulus environment is updated. This is closely linked to concepts of orienting/surprise and predictive coding (Eichele et al., 2005). A later aspect of P3, the late positive complex (LPC) is thought to more closely represent working memory and response selection (Donchin, 1981; Donchin and Coles, 1998, 2010; Polich, 2007). Contingent upon this, the P3 is also sensitive to changes in conflict and control (Clayson and Larson, 2011a,b). Due to the ability of children with TS to react to the presence of internal cues (premonitory urges) we expected a superior function of this electrophysiological correlate for performance monitoring.

After errors, the error-related negativity (ERN) and error positivity (Pe) are detectable. The ERN arises immediately after error commission (Debener et al., 2005; Larson et al., 2014) and reflects automatic error detection in the mesial frontal cortex. Individuals with several neuropsychiatric disorders, including adolescents with ADHD (Albrecht et al., 2008) show a reduction of this early negativity. Finally, the ERN is followed by the Pe, a P3-like positive deflection, emerging approximately 300 ms after incorrect responses and is associated with evaluation and awareness, as well as the salience of errors. It is important to note here that the ERN is not fully established before adolescence and was therefore not focus in our study, whereas the Pe amplitude does not appear to change much with age (Davies et al., 2004; Ladouceur et al., 2007; Wiersema et al., 2007; Brydges et al., 2013; Tamnes et al., 2013; Dupuis et al., 2015).

To our knowledge, no prior ERP study has used this type of Flanker task in children with TS. However, one behavioral study reported that children with TS performed slightly less accurately on incompatible trials (Crawford et al., 2005). Only few ERP studies overall have included children with TS, mainly auditory oddball paradigms have been used with variable results (Van Woerkom et al., 1994; Oades et al., 1996; Zhu et al., 2006). A recent study using a Go/Nogo paradigm (Shephard et al., 2015) did not report significant differences in the ERP in children with TS compared with controls. However, here two distinct subcomponents of a P3 can be appreciated, which each show a differential amplitude modulation between the groups, where indeed the TS group grand average has highest amplitudes during an earlier subcomponent (Shephard et al., 2015) and thus add to motivate further study of this component in children with TS. Interestingly, this component seems reduced in children with ADHD (Albrecht et al., 2008). These independent observations motivate the focus on P3 in the current analysis.

A larger amount of data exists from children with ADHD, indicating either non-different or reduced N2, P3, ERN, and Pe amplitudes compared with controls (for an overview, see Barry et al., 2003; Johnstone et al., 2013). We aimed at investigating electrophysiological measures in the Flanker task related to attention, stimulus evaluation, conflict and control in medication-naïve children with TS, compared with medication-naïve children with ADHD and controls, primarily in the N2-P3 latency range and the post-response ERN-Pe. We hypothesized that participants with TS would show a typical or enhanced performance and ERP amplitudes similar to control participants, whereas participants with ADHD would show impaired performance (Willcutt et al., 2005; Mazaheri et al., 2014) and reduced ERP amplitudes. Due to the limited ERP-literature on children with TS we do not only present hypothesized effects but all components involved in the Flanker task for reference and discovery of knowledge in the field of child psychopathology (Loo et al., 2015). Comparisons between groups should not be limited to measurement of one component to ensure that significant differences between groups are not ceiling effects transporting smaller differences from one component to the next until adding up to a significant difference (Picton et al., 2000).

We focus on performance monitoring in children with TS, and, due to ADHD being a frequent comorbidity, we also included participants with TS and comorbid ADHD. This group is compared with children with ADHD, and a group of typically developing children. This allows to leverage the impact of comorbid ADHD in combination with TS, as well as to measure the specific contribution of TS on our main outcome variables. The recent attempt to collect data across the boundaries of diagnostic entities calls for the inclusion of contrastgroups to allow differentiating characteristics found in individuals with a specific disorder from more general markers present across conditions (Cuthbert, 2014).

## Materials and methods

### Participants

One hundred and two participants were recruited for a prospective longitudinal study of children with ADHD, Tourette syndrome, and control children aged 8–12 years. Participants with ADHD and TS were recruited from the Department of Child and Adolescent Psychiatry, Haukeland University Hospital, and from outpatient clinics in the greater Bergen area in the Hordaland County, Norway. Controls were recruited from local schools in the same geographic regions. The Regional Ethics Committee approved the study, and written consent in accordance with the Declaration of Helsinki was obtained from all parents. The diagnostic procedure consisted of a semi-structured interview, the K-SADS (Kiddie-Schedule for Affective Disorders and Schizophrenia for School-Aged Children; Kaufman et al., 1997); the Children Gobal Assessment Scale (CGAS; Shaffer et al., 1983), and the DuPaul ADHD-Rating Scale (ADHD-RS; Dupaul et al., 1998), along with a best estimate consensus procedure that considered all available study material (Leckman, 2002). TS and ADHD diagnoses, respectively, met the criteria set in Diagnostic and Statistical Manual of Mental Disorders, fourth edition (DSM-IV; American Psychiatric Association, 1994). Tic symptoms were measured with the Yale Global Tic Severity scale (YGTSS; Leckman et al., 1989). All children were native Norwegian speakers of Caucasian origin, were medication-naïve and had no prior treatment for ADHD. Exclusion criteria for the control group were a lifetime history of Tic disorder, Obsessive compulsive disorder (OCD), ADHD, or a current DSM-IV axis I disorder. Additional exclusion criteria for all groups were epilepsy, head trauma with loss of consciousness, autism spectrum disorder, prematurity (gestational age <36 weeks), or a full scale intelligence quotient (FSIQ) below 75, measured by the Wechsler Intelligence Scale for Children-IV (Wechsler, 2003). Children with ADHD had a diagnosis of ADHD, combined type (*n* = 25), inattentive type (*n* = 11) or hyperactive type (*n* = 3). Within the study groups, the following comorbid disorders were present: oppositional defiant disorder (ODD; ADHD *n* = 17, TS *n* = 7), and three children with ADHD also had conduct disorder (CD), chronic and transient tics (ADHD *n* = 3), OCD (TS *n* = 2), and elimination disorder (ADHD *n* = 4, TS *n* = 3, controls *n* = 2). Moreover, several children fulfilled criteria for phobia (ADHD *n* = 7, TS *n* = 3, control *n* = 1), separation anxiety (ADHD *n* = 6, TS *n* = 1) and general anxiety (ADHD *n* = 3, TS *n* = 1). Thirteen children with TS had an additional ADHD diagnosis (ADHD combined type *n* = 7, ADHD inattentive type *n* = 6), 1 of these had an additional OCD diagnosis.

### Experimental design

After instruction and training, participants performed a modified visual Eriksen-Flanker task implemented in E-prime 2 (Psychology Software Tools, Inc., Pittsburgh, PA, USA). Participants were instructed to fixate a dot presented in the center of a PC screen. Trials began with the presentation of 6 horizontal flanker arrows appearing below fixation. Participants should respond as fast as possible, and as accurate as possible with either a left or a right mouse button press following the direction of a central target arrow that appeared after 100 ms, pointing either into the same direction as the flanker arrows in compatible trials (<<< < <<<, >>> > >>>) or in the opposite direction in incompatible trials (<<< > <<<, >>> < >>>). The target- and flanker-arrows remained on screen until a response was registered. Trials were terminated by the motor response and followed by an 800-ms interval before onset of the next trial. Stimuli were presented in two blocks with 200 trials that were pseudorandomized separately for each participant. The overall probability of compatible and incompatible trials, as well as left and right responses were kept at 0.5. Performance feedback was given during the experiment when responses were erroneous or slower than an adaptive individual threshold value (mean response time plus 1.5 standard deviations (SD).

### EEG acquisition

EEG was recorded continuously in an electromagnetically shielded chamber. Data were sampled at 1000 Hz frequency with a time-constant of 10 s and a high cutoff at 250 Hz with Brain Amp amplifiers (BrainProducts, Munich, Germany). An elastic cap containing 34 Ag/AgCl electrodes placed at Fp1, Fp2, F7, F3, Fz, F4, F8, FT9, FC5, FC1, FC2, FC6, FT10, T7, C3, Cz, C4, T8, TP9, CP5, CP1, CP2, CP6, TP10, P7, P3, Pz, P4, P8, PO9, O1, O2, PO10, Iz was used. Channels were referenced to Fz. Vertical eye movements were recorded with a bipolar derivation between Fp1 and an additional electrode placed below the left eye, horizontal eye movement were recorded with a bipolar derivation between F7 and F8. Additionally, electrocardiogram was monitored. Impedances were kept below 10 kΩ.

### EEG processing

We preprocessed the EEG in Matlab (Mathworks, Natick, MA, USA) using the EEGLAB toolbox (Delorme and Makeig, 2004) and in-house scripts.

The continuous EEG data were resampled to 500 Hz. The data were then re-referenced to common average reference, and filtered from 0.5 to 45 Hz using a finite impulse response filter generated with the firfilt plugin (Widmann, 2006).

For artifact removal/reduction, the data were segmented into stimulus-locked (−0.5 to +1 s), and response-locked epochs (−1 to 0.5 s). The prestimulus period was used as baseline for both epochs. Epochs were excluded when exceeding a ±300 μV amplitude criterion. The remaining epochs were sorted using a summary score of root mean square amplitude across all channels and time points, spatial SD, power spectrum ratio between low and high frequencies, skewness and kurtosis, normalized to unit variance across epochs. Only epochs within ±1 SD were retained for further analysis. These epochs were concatenated and subjected to temporal independent component analysis (ICA) using the infomax algorithm (Bell and Sejnowski, 1995), and 32 components were estimated. We used spatial templates to identify horizontal and vertical eye movements and ECG artifacts, and removed these automatically (Viola et al., 2009). Following the rationale presented in COMPASS (Wessel and Ullsperger, 2011), we assumed that components of interest were broad, dipolar topographies with time-locked event-related responses, and we therefore generated scores based on the spatial smoothness of the component scalp maps and the root mean square of the event related average, and retained the top 15 components. These were then visually cross-checked, and components reminiscent of artifacts were marked. Between 10 and 15 components were kept and back-projected in this manner.

### Averaging and data extraction

**Figure 1:**
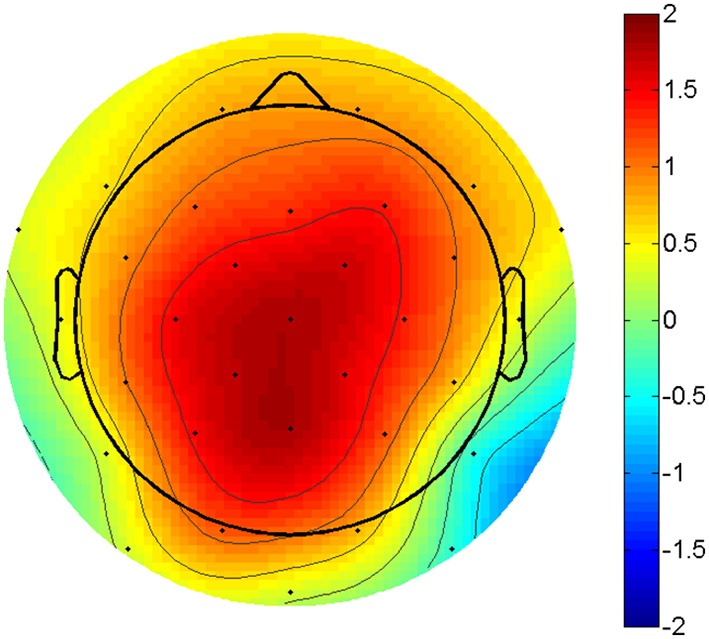
**P3 amplitude difference**. Topographic plot of the amplitude difference between compatible and incompatible outcomes at 300 ms post stimulus. Scaling from -2 to 2 μV. Voltage maximum is at centro-parietal midline sites.

We sorted compatible, incompatible and erroneous trials and visually inspected the grand averaged data across all participants to generate ERP for further testing. Upon inspection of grand average ERP data and difference waves, we found that conditional effects on several components were consistently expressed around Cz/Vertex, which is in line with other work in this age group (Cycowicz, 2000; Stige et al., 2007). We therefore used regional averaging, for spatial data reduction, and controlling for inter-individual variability (Handy, 2005). This provides a better fit to the statistical models by collapsing together electrodes that commonly covary, in the same way that adding a spatial factor would do, however without complicating the analysis by additional terms of interaction. Moreover, it helps to control for variability (as seen in different age groups e.g., Cycowicz, 2000; Davies et al., 2004; Brydges et al., 2013) over locations by averaging across locations. This method addresses the objection to the large degrees of freedom that multiple electrode readings afford (Handy, 2005). We selected a central region of interest containing FC1, FC2, Cz, CP1, and CP2 with clear N1 (108 ms), P2 (196 ms), P3 (320 ms), and LPC (598 ms) waveforms in the stimulus-locked average, as well as LPC (−82 ms) in the response-locked average, and a clear modulation between outcomes (see Figure 1). ERN was identified as the first post-response negativity maximal on erroneous trials. The early positivity is defined as the first positive wave post-response—this common post-response component is labeled P2 or P90 elsewhere (Brunia and Van Boxtel, 2000). Error response generated an additional broad positivity Pe with peak latency at 268 ms post-response. Because latency jitter in ERP components between trials, especially in children, and peak amplitudes can be influenced by group differences in signal-noise-ratio, analyses of mean amplitudes were chosen (Luck, 2005). Amplitudes were extracted from 40 ms long windows centered on the grand average peak latency and were used for testing of group differences.

### Statistics

Statistics were performed in Matlab and Statistica (Statsoft, Tulsa, OK, USA). Repeated measure analyses were conducted to test outcome effects in the behavioral and the ERP data (congruent vs. incongruent vs. error trials) and “group.” Additional univariate Analysis of Covariance (ANCOVA) were conducted for behavioral measures and ERP components as dependent variable, group as categorical factor and covariates as continuous predictors to test group differences. Significant or trend-significant effects were followed-up with additional *post-hoc* tests. All statistics were considered significant at *p* < 0.05. The effect size indicator partial eta squared ($\eta_{p}^{2}$) is reported for each significant/trend-significant statistical comparison as a measure of the strength of the effect, with of 0.01 representing a small effect, 0.06 a medium effect, and 0.14 a large effect (Cohen, 1988). To demonstrate the adequacy of pooling children with TS with and without comorbid ADHD, we also performed ANCOVAs with four groups, separating TS only and TS+ADHD, control group, ADHD, with the main behavioral and ERP result.

Response times (RT) and response accuracy (RACC) averages were generated for all possible outcomes. Premature responses faster than 200 ms and slow responses >2000 ms were not considered in the averages. RTs were analyzed with covariates:

Age: Because of substantial speeding of RT, and improvement of accuracy with age across the entire sample regardless of group, all analyses included age as a covariate.

FSIQ: We decided to analyze the behavioral data with FSIQ as covariate for the sake of consistency across behavioral- and ERP analyses. This appears to be the most sound practice in our case, however see relevant publications for a discussion on this issue (Willcutt et al., 2005; Dennis et al., 2009).

ERP components were analyzed with covariates:

Age: Groups did not differ in mean age. However, to control within group variation of electrophysiological measures we followed current guidelines (Picton et al., 2000). Age in particular influences many features in the EEG, resulting also in prominent maturational changes of ERP amplitudes and latencies (Davies et al., 2004; Wiersema et al., 2007; Brydges et al., 2013; Rojas-Benjumea et al., 2015).

FSIQ: Earlier research has also shown that IQ differences account for variability of ERP measures. We therefore decided to include IQ as a covariate in line with other studies in the field (Pelosi et al., 1992; Deary and Caryl, 1997; Jausovec and Jausovec, 2000; Ramchurn et al., 2014).

RT/ IIV: Response times and their variability substantially affect ERP features (Eichele et al., 2010b). This is partly due to task-induced amplitude modulation, and partly nuisance variability due to spatio-temporal overlap of stimulus and response-related components, see also (Ramchurn et al., 2014).

RACC: Average accuracy provides a gross measure of the effort that an individual invests in a task, therefore adjusting for ACC is useful to account for state and trait factors not specifically related to diagnosis/group.

ADHD symptom scores were included initially as a covariate in the statistical models for the behavioral and the ERP correlates, but proved non-significant and were subsequently removed from both models. Pairwise correlations were used to further investigate significant effects of the group factor and covariates. To test for post-error slowing (PES) and to compensate for confounders, we conducted a pairwise comparison of post-error and pre-error trials around each error (Dutilh et al., 2012) followed by an ANCOVA, including the covariates age and FSIQ.

### Behavioral characteristics

Data from two participants (with ADHD and with TS/ADHD, respectively) were discarded due to excessive EEG artifact, data from another participant (ADHD) were discarded due to performance on chance level, data from 99 participants thus were included, 39 children with a diagnosis of ADHD, 25 children with TS (11 TS “only” and 14 TS+ADHD), and 35 typically developing children. Children's age ranged from 8 to 12 years (*M* = 10.05; SD ± 1.21), 64 participants were boys and groups did not differ for age or sex. 15 participants were left-handed. Groups differed in FSIQ, similar to findings reported in other studies (Bornstein, 1991; Ozonoff et al., 1998; Baym et al., 2008; Debes et al., 2011), and FSIQ was employed as a covariate. Groups also differed in ADHD-RS total values. Current tic severity in the TS group was 11.3 ± 3.34 for motor and 8.00 ± 4.83 for vocal tics, and lifetime worst ever score 15.68 ± 3.44 for motor and 11.95 ± 5.0 for vocal tics (Table 1).

**Table 1:** **Sample characteristics**.

	**Controls Mean ± *SD***	**ADHD Mean ± *SD***	**TS Mean ± *SD***	**Statistics**
FSIQ	105.82 ± 1.68	91.71 ± 1.59	97.96 ± 1.99	$F_{\left(2,\;96\right)}=18.51,p<0.001,\eta_{p}^{2}=0.28$
Age (years)	10.04 ± 0.21	10.18 ± 0.19	9.87 ± 0.24	*F*_(2, 96)_ = 0.49, n.s.
Sex (% male)	57.14	69.23	68	χ2 = 1.34, n.s.
Handeness (% right handed)	91.43	84.62	76	χ2 = 2.7, n.s.
ADHD-RS total score	2.91 ± 1.33	30.73 ± 1.26	22.12 ± 1.57	$F_{\left(2,\;96\right)}=117.09,p<0.001,\eta_{p}^{2}=0.62$

ADHD, attention-deficit/hyperactivity disorder; TS, Tourette syndrome; FSIQ, full scale intelligence quotient; ADHD-RS, attention-deficit/hyperactivity disorder rating scale; SD, standard deviation.

## Results

### Behavioral performance

We observed no significant differences between groups for premature responses, but a significant effect of FSIQ, with a weak correlation where lower FSIQ correlated with more premature responses (*r* = −0.29). Slow responses were more frequent in all groups compared with fast responses, also with a significant FSIQ effect, with correlations for lower FSIQ predicting more frequent slower responses (*r* = −0.34) and age (*r* = −0.38). (Table 2).

**Table 2:** **Behavioral performance**.

	**Controls**	**ADHD**	**TS**	**Repeated Measure ANOVA**			**ANCOVA**	
	**Mean ±*SD***	**Mean ±*SD***	**Mean ±*SD***	**Outcome**	**Group**	**Outcome × group**	**Group**	**Covariates**
RT CC (ms)	646.44 ± 19.69	677.37 ± 18.43	652.99 ± 21.26	$F_{\left(2,\;192\right)}=136.9,p<0.001,\eta_{p}^{2}=0.59$	$F_{\left(2,\;96\right)}=2.85,p=0.06,\eta_{p}^{2}=0.06$	*F*_(4, 192)_ = 1.14, n.s.	*F*_(2, 94)_ = 0.65, n.s.	FSIQ $F_{\left(1,\;94\right)}=11.77,p<0.001,\eta_{p}^{2}=0.11$; Age $F_{\left(1,\;94\right)}=64.68,p<0.001,\eta_{p}^{2}=0.41$
RT IC (ms)	773.11 ± 25.73	840.70 ± 24.01	789.58 ± 27.77				*F*_(2, 94)_ = 1.77, n.s.	FSIQ $F_{\left(1,\;94\right)}=10.19,p<0.01,\eta_{p}^{2}=0.09$; Age $F_{\left(1,\;94\right)}=61.04,p<0.001,\eta_{p}^{2}=0.39$
RT error (ms)	624.42 ± 29.70	644.70 ± 27.79	627.28 ± 32.06				*F*_(2, 94)_ = 0.13, n.s.	FSIQ $F_{\left(1,\;94\right)}=7.5,p<0.01,\eta_{p}^{2}=0.07$; Age $F_{\left(1,\;94\right)}=34.06,p<0.001,\eta_{p}^{2}=0.27$
IIV CC (ms)	209.48 ± 10.98	245.21 ± 10.27	231.66 ± 11.85	$F_{\left(2,\;192\right)}=25.46,p<0.001,\eta_{p}^{2}=0.21$	$F_{\left(2,\;96\right)}=5.68,p<0.01,\eta_{p}^{2}=0.11$	*F*_(4, 192)_ = 0.71, n.s.	*F*_(2, 94)_ = 2.46, n.s.	FSIQ $F_{\left(1,\;94\right)}=14.43,p<0.001,\eta_{p}^{2}=0.13$; Age $F_{\left(1,\;94\right)}=32.72,p<0.001,\eta_{p}^{2}=0.26$
IIV IC (ms)	245.13 ± 12.95	261.21 ± 12.12	266.46 ± 13.98				*F*_(2, 94)_ = 0.66, n.s.	FSIQ $F_{\left(1,\;94\right)}=12.66,p<0.001,\eta_{p}^{2}=0.12$; Age $F_{\left(1,\;94\right)}=22.48,p<0.001,\eta_{p}^{2}=0.19$
IIV error (ms)	271.01 ± 20.41	309.89 ± 19.09	290.70 ± 22.03				*F*_(2, 94)_ = 0.83, n.s.	FSIQ $F_{\left(1,\;94\right)}=7.75,p<0.01,\eta_{p}^{2}=0.08$; Age $F_{\left(1,\;94\right)}=26.73,p<0.001,\eta_{p}^{2}=0.22$
Compatible errors(%)	6.57 ± 1.07	7.85 ± 1.01	9.32 ± 1.26	$F_{\left(1,\;96\right)}=142.14,p<0.001,\eta_{p}^{2}=0.6$	$F_{\left(2,\;96\right)}=2.46,p=0.09,\eta_{p}^{2}=0.05$	$F_{\left(2,\;96\right)}=3.01,p=0.06,\eta_{p}^{2}=0.06$	*F*_(2, 92)_ = 1.47, n.s.	FSIQ $F_{\left(1,\;94\right)}=7.12,p<0.01,\eta_{p}^{2}=0.07$
Incompatible errors (%)	18.96 ± 2.37	20.65 ± 2.21	21.78 ± 2.56				*F*_(2, 92)_ = 0.32, n.s.	FSIQ $F_{\left(1,\;92\right)}=9.53,p<0.01,\eta_{p}^{2}=0.003$
Responses < 200 ms (n)	6.79 ± 2.64	4.77 ± 2.47	9.01 ± 2.85				*F*_(2, 94)_ = 0.63, n.s.	FSIQ $F_{\left(1,\;94\right)}=8.35,p<0.01,\eta_{p}^{2}=0.08$
Responses >2000 ms (n)	12.49 ± 3.59	21.65 ± 3.36	11.73 ± 3.87				*F*_(2, 94)_ = 2.29, n.s.	FSIQ $F_{\left(1,\;94\right)}=7.22,p<0.01,\eta_{p}^{2}=0.07$; Age $F_{\left(1,\;94\right)}=23.22,p<0.001,\eta_{p}^{2}=0.19$
PES (ms)	79.47 ± 20.71	87.11 ± 19.38	36.73 ± 22.36				*F*_(2, 94)_ = 1.67, n.s.	
Overall RT (ms)	695.49 ± 22.16	735.80 ± 20.73	690.72 ± 23.92				*F*_(2, 94)_ = 1.21, n.s.	FSIQ $F_{\left(1,\;94\right)}=8.01,p<0.01,\eta_{p}^{2}=0.08$; Age $F_{\left(1,\;94\right)}=56.71,p<0.001,\eta_{p}^{2}=0.38$
Overall IIV (ms)	255.11 ± 11.26	286.59 ± 10.53	270.25 ± 12.15				*F*_(2, 94)_ = 1.8, n.s.	FSIQ $F_{\left(1,94\right)}=17.21,p<0.001,\eta_{p}^{2}=0.15$; Age $F_{\left(1,\;94\right)}=38.29,p<0.001,\eta_{p}^{2}=0.29$
Overall RACC (%)	86.68 ± 1.63	86.54 ± 1.52	84.64 ± 1.75				*F*_(2, 92)_ = 0.25, n.s.	FSIQ $F_{\left(1,\;92\right)}=10.05,p<0.01,\eta_{p}^{2}=0.10$

CC, compatible correct; IC, incompatible correct; PES, post-error slowing; RT, reaction time; IIV, intraindividual variability; RACC, response accuracy; ANCOVA, analysis of covariance; ANOVA, analysis of variance, ADHD, attention-deficit/hyperactivity disorder; TS, Tourette syndrome; SD, standard deviation; n.s., not significant; Responses >2000 ms are considered equivalent to omissions, Responses < 200 ms are considered false alarms.

### Reaction times

A Repeated Measure Analysis revealed a typical RT pattern for the Flanker task with fast RT in compatible (CC) responses, slower incompatible (IC) responses and faster RT in erroneous trials in all three groups, and trend-significant group differences across all three outcomes [*F*_(2, 96)_ = 2.85, *p* = 0.06, $\eta_{p}^{2}=0.06$], without significant interactions of outcome-by-group. *Post-hoc* assessment revealed trend-significant differences for CC responses (*p* = 0.07) and erroneous responses (*p* = 0.08) between controls and ADHD and a significant difference in IC trials (*p* < 0.01) between controls and ADHD. No differences between children with TS and controls.

When controlling for covariates in a follow-up ANCOVA, the CC, IC or erroneous RTs did not differ between groups (Table 2).

### Response accuracy

Errors were defined as incorrect key presses to compatible and incompatible trials. As expected, significantly more errors occurred to incompatible than compatible trials [*F*_(1, 96)_ = 142.14, *p* < 0.001, $\eta_{p}^{2}=0.6$]. A repeated measure analysis revealed a trend-significant group difference [*F*_(2, 96)_ = 2.46, *p* = 0.09, $\eta_{p}^{2}=0.05$] and a trend-significant outcome-by-group difference [*F*_(2, 96)_ = 3.01, *p* = 0.06, $\eta_{p}^{2}=0.06$] which was due to higher incompatible error rates in children with ADHD (*p* < 0.01) and TS (*p* < 0.05) than controls.

After controlling for covariates (ANCOVA), groups did not differ in in error rates for either CC or IC responses, or for overall RACC with a significant effect of FSIQ (Table 2).

### Post error slowing

ANCOVA for PES yielded no significant group differences (Table 2).

### Intraindividual variability

A repeated measure analysis of IIV showed smaller IIV for compatible trials, larger IIV in incompatible trials and largest IIV in erroneous trials, and significant group differences across all three outcomes [*F*_(2, 96)_ = 5.68, *p* < 0.01, $\eta_{p}^{2}=0.11$]. No significant interaction for outcome-by-group was found.

When controlling for covariates in the follow-up ANCOVA groups did not differ with respect to IIV, but the relevant covariates FSIQ and age reached significance in the overall IIV, as well as in the separate CC, IC and error trials (Table 2).

### Electrophysiological results

**Figure 2:**
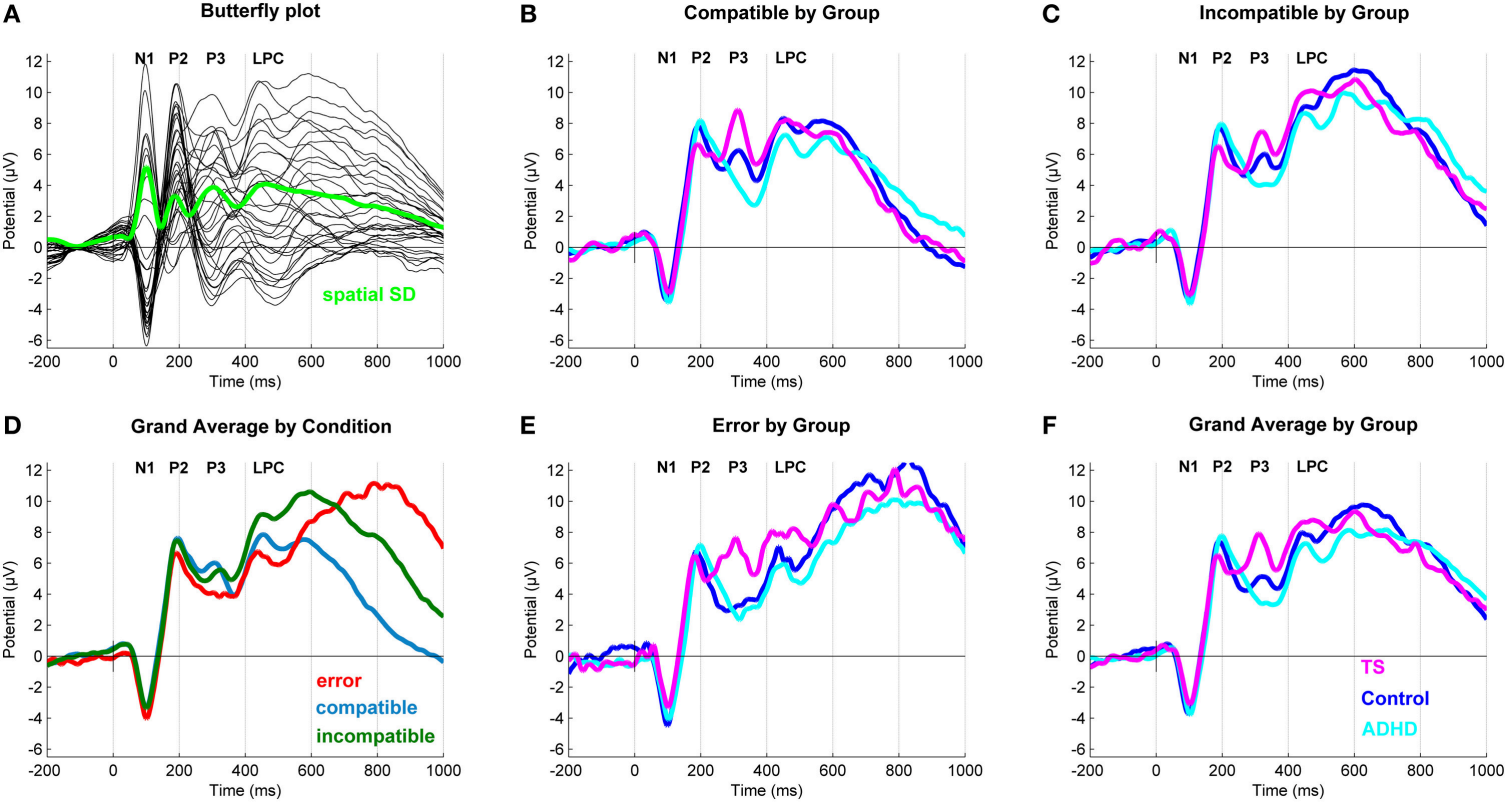
**Stimulus-locked event-related potentials (ERP). (A)** Butterfly plot. Topographical distribution of the P3 component across outcomes. **(D)** Grand average ERP by Outcome at a central region of interest for compatible (blue), incompatible (green) and erroneous (red) trials. **(B,C,E)** Grand average ERPs at a central region of interest with a N1 (108 ms), P2 (196 ms), P3 (320 ms), and a Late Positive Component (598 ms) for compatible, incompatible and erroneous trials. **(F)** Grand average ERP by Group. Group differences for Tourette syndrome (TS) (pink), attention-deficit/hyperactivity disorder (ADHD) (turquoise) and control children (blue) at a central region of interest.

**Figure 3:**
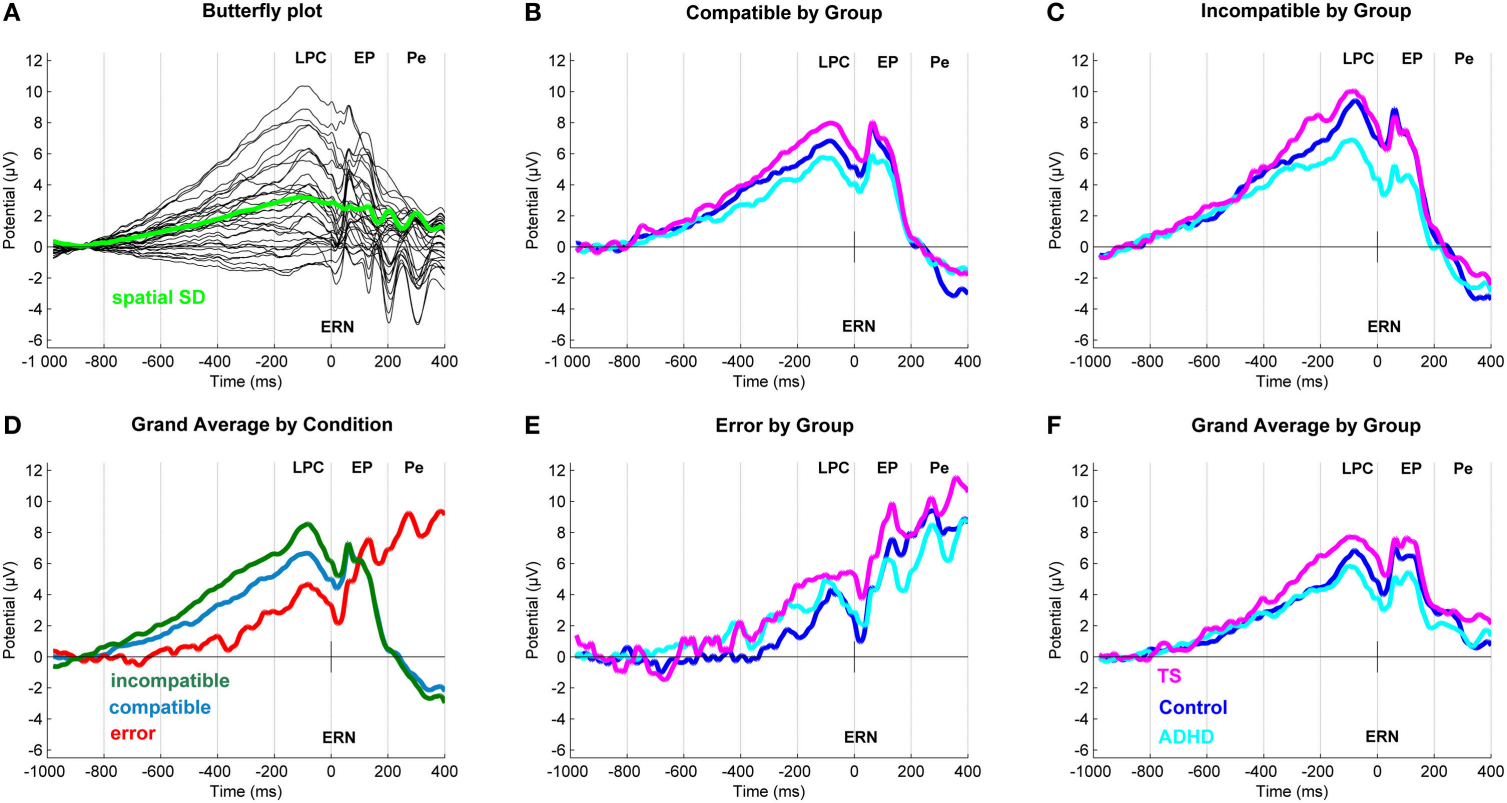
**Response-locked event-related potentials (ERP). (A)** Butterfly plot. Topographical distribution of the Late Positive Component across outcomes. **(D)** Grand average ERP by Outcome at a central region of interest for compatible (blue), incompatible (green) and erroneous (red) trials. **(B,C,E)** Linked mastoid referenced grand average ERPs at a central region of interest with a Late Positive Component (−80 ms) and early positivity (60 ms). In erroneous trials (bottom middle) also seen an ERN (20 ms) and a Pe (268 ms). **(F)** Grand average ERP by Group. Group differences for Tourette syndrome (TS) (pink), attention-deficit/hyperactivity disorder (ADHD) (turquoise) and control children (blue) at a central region of interest.

After inspection of the grand averages of the stimulus-locked (Figure 2) and response-locked (Figure 3) ERP data, we conducted repeated measure analyses for the components separately to test the presence of the typical compatibility/conflict effects considering the factors “outcome,” “group” and the “outcome x group” interaction. We observed significant “outcome” effects for the stimulus-locked P3 and LPC and the response-locked LPC, ERN and Pe. Trend-significant effects of “outcome” were seen in the P2, no significant effects of outcomes were seen for N1 and response-locked early positivity. We also did observe “group” and “outcome x group” effects, which were followed-up by appropriate ANCOVA designs controlling for confounds (Tables 3, 4).

**Table 3:** **Stimulus-locked ERP amplitudes**.

	**Controls**	**ADHD**	**TS**	**Repeated Measure ANOVA**			**ANCOVA**	
**Stimulus-locked**	**Mean ±*SD* (μV)**	**Mean ±*SD* (μV)**	**Mean ±*SD* (μV)**	**Outcome**	**Group**	**Outcome x Group**	**Group**	**Covariates**
**N1 (108 ms)**								
Compatible	−2.72±0.70	−3.04±0.65	−2.42±0.75	*F*_(2, 192)_ = 1.89, n.s.	*F*_(2, 96)_ = 0.22, n.s.	*F*_(4, 192)_ = 0.43, n.s.	*F*_(2, 91)_ = 0.19, n.s.	RT $F_{\left(1,\;91\right)}=5.95,p=0.02,\eta_{p}^{2}=0.06$;IIV $F_{\left(1,\;91\right)}=7.06,p<0.01,\eta_{p}^{2}=0.07$
Incompatible	−2.49±0.68	−3.47±0.63	−2.36±0.73				*F*_(2, 91)_ = 0.74, n.s.	
Error	−3.07±0.90	−4.03±0.84	−2.79±0.97				*F*_(2, 91)_ = 0.5, n.s.	
Mean	−2.80±0.69	−3.44±0.65	−2.57±0.75					
**P2 (196 ms)**								
Compatible	7.19 ± 0.91	7.05 ± 0.84	6.36 ± 0.96	$F_{\left(2,\;192\right)}=2.69,p=0.06,\eta_{p}^{2}=0.03$	*F*_(2, 96)_ = 0.21, n.s.	*F*_(4, 192)_ = 0.35, n.s.	*F*_(2, 91)_ = 0.23, n.s.	
Incompatible	7.23 ± 0.98	6.73 ± 0.92	6.33 ± 1.05				*F*_(2, 91)_ = 0.19, n.s.	
Error	6.53 ± 1.06	5.82 ± 0.99	5.99 ± 1.13				*F*_(2, 91)_ = 0.11, n.s.	
Mean	6.89 ± 0.91	6.64 ± 0.85	6.19 ± 0.97					
**P3 (320 ms)**								
Compatible	5.26 ± 1.04	4.70 ± 0.93	8.69 ± 1.07	$F_{\left(2,\;192\right)}=11.71,p<0.001,\eta_{p}^{2}=0.11$	$F_{\left(2,\;96\right)}=3.61,p=0.03,\eta_{p}^{2}=0.07$	$F_{\left(4,\;192\right)}=2.21,p=0.06,\eta_{p}^{2}=0.04$	$F_{\left(2,\;91\right)}=4.62,p=0.01,\eta_{p}^{2}=0.09$	RACC $F_{\left(1,\;91\right)}=4.43,p=0.04,\eta_{p}^{2}=0.05$; IIV $F_{\left(1,\;91\right)}=5.21,p=0.03,\eta_{p}^{2}=0.05$
Incompatible	4.60 ± 1.03	4.46 ± 0.97	7.61 ± 1.12				F${}_{\left(2,91\right)}=2.82,p=0.06,\eta_{p}^{2}=0.06$	
Error	2.84 ± 1.15	2.89 ± 1.07	7.47 ± 1.23				$F_{\left(2,\;91\right)}=5.17,p<0.01,\eta_{p}^{2}=0.10$	
Mean	4.09 ± 0.99	4.22 ± 0.93	7.81 ± 1.06					
**LPC (598 ms)**								
Compatible	7.12 ± 0.96	7.78 ± 0.89	8.46 ± 1.02	$F_{\left(2,\;192\right)}=14.35,p<0.001,\eta_{p}^{2}=0.13$	*F*_(2, 192)_ = 0.61, n.s.	*F*_(4, 192)_ = 0.48, n.s.	*F*_(2, 91)_ = 0.45, n.s.	RACC $F_{\left(1,\;91\right)}=3.96,p=0.05,\eta_{p}^{2}=0.04$
Incompatible	8.11 ± 1.03	8.89 ± 0.96	10.86 ± 1.11				*F*_(2, 91)_ = 1.79, n.s.	IIV $F_{\left(1,\;91\right)}=9.01,p<0.01,\eta_{p}^{2}=0.09$
Error	5.95 ± 1.39	5.73 ± 1.31	8.13 ± 1.50				F_(2, 91)_ = 0.88, n.s.	
Mean	6.83 ± 1.00	7.72 ± 0.94	9.07 ± 1.08					

ERP, event-related potentials; SD, standard deviation; ANOVA, analysis of variance; ADHD, attention-deficit/hyperactivity disorder; TS, Tourette syndrome; LPC, Late positive component; n.s., not significant.

**Table 4:** **Response-locked ERP amplitudes**.

	**Controls**	**ADHD**	**TS**	**Repeated measure ANOVA**			**ANCOVA**	
**Response-locked**	**Mean ±*SD* (μV)**	**Mean ±*SD* (μV)**	**Mean ±*SD* (μV)**	**Outcome**	**Group**	**Outcome × Group**	**Group**	**Covariates**
**LPC (**−**82 ms)**								
Compatible	6.37 ± 0.88	5.88 ± 0.82	8.14 ± 0.93	$F_{\left(2,\;192\right)}=26.92,p<0.001,\eta_{p}^{2}=0.22$	*F*_(2, 96)_ = 1.21, n.s.	*F*_(4, 192)_ = 2.01, *p* = 0.09, η${}_{p}^{2}=0.04$	*F*_(2, 91)_ = 1.84, n.s.	RACC *F*_(1, 91)_ = 8.21, *p* < 0.01, η${}_{p}^{2}=0.08$; RT *F*_(1, 91)_ = 16.46, *p* < 0.001, η${}_{p}^{2}=0.15$
Incompatible	7.46 ± 0.87	8.24 ± 0.82	10.23 ± 0.94				*F*_(2, 91)_ = 2.5, *p* = 0.08, η${}_{p}^{2}=0.05$	
Error	4.41 ± 1.25	4.34 ± 1.17	5.29 ± 1.34				*F*_(2, 91)_ = 0.17, n.s.	
Mean	6.09 ± 0.86	6.19 ± 0.81	7.79 ± 0.92					
**ERN (20 ms)**								
Compatible	4.55 ± 0.89	4.25 ± 0.84	5.71 ± 0.96	*F*_(2, 192)_ = 13.04, *p* < 0.001, η${}_{p}^{2}=0.12$	*F*_(2, 96)_ = 1.46, n.s.	*F*_(4, 192)_ = 2.09, *p* = 0.08, η${}_{p}^{2}=0.04$	*F*_(2, 91)_ = 0.74, n.s.	RT *F*_(1, 91)_ = 16.45, *p* < 0.001, η${}_{p}^{2}=0.15$; Age *F*_(1, 91)_ = 6.57, *p* = 0.01, η${}_{p}^{2}=0.07$
Incompatible	4.85 ± 0.90	5.28 ± 0.85	6.52 ± 0.98				*F*_(2, 91)_ = 0.87, n.s.	
Error	1.93 ± 1.50	1.73 ± 1.40	4.24 ± 1.61				*F*_(2, 91)_ = 0.84, n.s.	
Mean	3.77 ± 0.96	3.80 ± 0.80	5.42 ± 1.03					
**EARLY POSITIVITY (62 ms)**								
Compatible	6.47 ± 0.96	6.02 ± 0.89	7.23 ± 1.02	*F*_(2, 192)_ = 1.72, n.s.	*F*_(2, 96)_ = 1.53, n.s.	*F*_(4, 192)_ = 1.69, n.s.	*F*_(2, 91)_ = 0.41, n.s.	RT *F*_(1, 91)_ = 14.17, *p* < 0.001, η${}_{p}^{2}=0.13$; Age *F*_(1, 91)_ = 4.35, *p* = 0.04, η${}_{p}^{2}=0.05$
Incompatible	5.80 ± 0.98	6.12 ± 0.93	7.49 ± 1.06				*F*_(2, 91)_ = 0.78, n.s.	
Error	5.12 ± 1.51	4.59 ± 1.41	7.15 ± 1.62				*F*_(2, 91)_ = 0.78, n.s.	
Mean	5.81 ± 1.02	5.61 ± 0.96	7.20 ± 1.09					
**Pe (268 ms)**								
Compatible	−0.07±0.99	−1.45±0.92	−0.88±1.05	*F*_(2, 192)_ = 115.16, *p* < 0.001, η${}_{p}^{2}=0.55$	*F*_(2, 96)_ = 0.31, n.s.	*F*_(4, 192)_ = 0.12, n.s.	*F*_(2, 91)_ = 0.44, n.s.	RT $F_{\left(1,\;91\right)}=8.58,p<0.01,\eta_{p}^{2}=0.08$; Age $F_{\left(1,\;91\right)}=4.27,p=0.04,\eta_{p}^{2}=0.04$
Incompatible	−1.13±0.94	−1.31±0.88	−0.99±1.01				*F*_(2, 91)_ = 0.03, n.s.	FSIQ $F_{\left(1,\;91\right)}=5.39,p=0.02,\eta_{p}^{2}=0.06$; RACC $F_{\left(1,\;91\right)}=8.84,p<0.01,\eta_{p}^{2}=0.09$
Error	9.31 ± 1.68	8.08 ± 1.56	10.42 ± 1.79				*F*_(2, 91)_ = 0.48, n.s.	
Mean	2.58 ± 1.01	1.93 ±.94	2.78 ± 1.08					

ERP, event-related potentials; SD, standard deviation; ANOVA, analysis of variance; ADHD, attention-deficit/hyperactivity disorder; TS, Tourette syndrome; LPC, Late positive component; ERN, error-related negativity; Pe, error positivity; n.s., not significant.

### Stimulus-locked ERPs (Table 3)

#### N1 (108 ms)

ANCOVA showed no group differences in compatible, incompatible, and error outcomes. A significant effect of RT and IIV was present in incompatible correct outcomes.

#### P2 (196 ms)

No group effects were found in ANCOVA for compatible, incompatible, and erroneous P2 amplitudes.

#### P3 (320 ms)

ANCOVA yielded a significant group effect for compatible correct [*F*_(2, 91)_ = 4.62, *p* = 0.01, $\eta_{p}^{2}=0.09$] and erroneous responses [*F*_(2, 91)_ = 5.17, *p* < 0.01, $\eta_{p}^{2}=0.10$]. Incompatible correct outcomes also approached significance [*F*_(2, 91)_ = 2.82, *p* = 0.06, $\eta_{p}^{2}=0.06$], and a significant effect of RACC and IIV was observed. A *post-hoc* assessment revealed that, P3 amplitudes across outcomes were higher in children with TS compared to both controls (*p* < 0.05), and those with ADHD (*p* < 0.05). No significant differences were found between participants with ADHD and controls.

#### LPC (600 ms)

ANCOVA showed no significant group difference in compatible correct outcomes, there was a significant effect of RACC. Similarly, no differences were present in incompatible correct outcomes, while a significant covariate-effect of IIV was present. No differences were found in erroneous LPC amplitudes.

### Response-locked ERPs (Table 4)

#### LPC (−80 ms)

ANCOVA showed no significant group differences in compatible and erroneous amplitudes. Incompatible amplitudes showed a trend-significant group effect [*F*_(2, 91)_ = 2.5, *p* = 0.08, $\eta_{p}^{2}=0.05$], with a significant effect of RACC and RT. *Post-hoc* tests showed higher amplitudes in TS vs. controls (*p* = 0.04), and a similar trend between TS and ADHD (*p* = 0.09), but no difference between controls and ADHD.

#### ERN (20 ms)

In this sample, we did not observe a distinct negative ERN in this age group, consistent with Davies (Davies et al., 2004). However, the most negative amplitudes during the post-response period were seen for erroneous trials, and a trend-significant outcome-by-group effect [*F*_(4, 192)_ = 2.09, *p* = 0.08, $\eta_{p}^{2}=0.04$]. However, this was due to higher incompatible amplitudes for controls than ADHD (*p* = 0.03) and similarly for TS compared to ADHD (*p* = 0.06), whereas no differences were seen between TS and controls. Note though that there is a substantial carry-over of the amplitude modulation from the preceding LPC into this time-window, especially for correct responses.

When controlling for covariates, ANCOVA showed no group differences in any trial outcome, whereas clear effects of age and RT were present for incompatible correct.

#### Early positivity (60 ms)

ANCOVA showed no significant group differences across outcomes. Significant effect of RT and age were present only for incompatible outcomes.

#### Pe (268 ms)

ANCOVA showed no group differences throughout. Incompatible ERPs showed a significant effect of RT and age, during erroneous trials with we saw a significant effect of FSIQ and RACC.

#### Correlation with symptoms

We found no robust correlations between behavioral or ERP measurements and YGTSS scores.

#### Grouping of children with TS only and with TS and comorbid ADHD

To demonstrate the adequacy of pooling children with TS with and without ADHD, we performed ANCOVAs with four groups, separating TS only and TS+ADHD, control group, ADHD, with the main behavioral and ERP result.

For RT, this analysis showed no group difference [*F*_(3, 93)_ = 1.21, *p* = 0.31, $\eta_{p}^{2}=0.03$]. Comorbid ADHD in the TS group resulted in marginally different RTs compared to TS only (*p* = 0.99) and controls (*p* = 0.78). Children with ADHD showed high RTs, but no significant differences to other groups (*p* > 0.2).

Also for the IIV, no group differences were found [*F*_(3, 93)_ = 1.21, *p* = 0.31, $\eta_{p}^{2}=0.03$] and a *post-hoc* comparison revealed no significant differences between the groups (all *p* ≥ 0.1).

For the P3 this analysis repeats a significant group difference [*F*_(3, 90)_ = 2.99, *p* = 0.04, $\eta_{p}^{2}=0.09$], and showed that comorbid ADHD resulted in marginally lower amplitude values in ERPs compared to participants with TS only without significant differences (*p* = 0.63). Children with TS+ADHD showed trends toward higher amplitudes than controls (*p* = 0.06) and children with ADHD (*p* = 0.06), whereas TS only had significantly larger amplitudes than ADHD (*p* = 0.03), and controls (*p* = 0.02).

Based on these additional analyses, the fact that the sample sizes in analyses of these subsamples are small, and the pattern of results redundant and the high clinical relevance of a comorbid group, we merged all participants with TS into one group.

## Discussion

This study investigated electrophysiological differences in a Flanker task in children with TS compared with children with ADHD and with typically developing children. We expected that children with TS would perform comparable or better than controls, whereas children with ADHD would show impairments of behavior and ERP measures.

Our results confirmed that children with TS performed behaviorally on the same level as control children. This finding is consistent with previous studies of behavioral performance (Serrien et al., 2005; Roessner et al., 2008; Eichele et al., 2010a; Greimel et al., 2011). In contrast to our expectations, the present study did not find group behavioral differences between children with ADHD and control children when controlling for relevant covariates, which stands in contrast to some previous findings (Albrecht et al., 2008), but not others (Johnstone and Galletta, 2013).

Children with TS, however, showed higher amplitudes in the stimulus-locked ERPs in the early P3 amplitude compared with children with ADHD and control children across task outcomes, which was sustained through the later positive complex. We speculate therefore, that this increased amplitude might reflect a process that may help children with TS to maintain their behavioral performance. The increase in P3 amplitude might reflect greater sustained effort in the TS group in processing the stimuli (Isreal et al., 1980; Luck, 2005) yielding in turn increased attentional resource allocation during stimulus processing. This is supported by the fact that the P3 in children with TS consistently higher across outcomes. Moreover, the increase in P3 in the TS group might indicate that children with TS displayed enhanced processes to update working memory. Together with the increase in the response-locked LPC amplitude, this might reflect an altered sustained attention/orienting pattern of whether the first decision of stimulus classification has led to appropriate steps of processing (Verleger et al., 2005) in children with TS.

Here, TS children show the largest peak of all groups in the earlier P3 subcomponent around 300–350 ms after flanker onset. A recent study using a Go/Nogo-paradigm (Shephard et al., 2015) in a similar cohort with a broader age range did not report differences in ERP correlates between children with TS and control children. The authors analyzed the P3 complex in a longer time-window from 300 to 650 ms. Interestingly, two distinct subcomponents of the P3 can be appreciated during this period, which each show a differential amplitude pattern between the groups, where indeed the TS group grand average has highest amplitudes during the earlier subcomponent (see Figure 3 in Shephard et al., 2015). Similarly, another experiment from the same group, the authors assessed goal directed learning and showed distinct P3 peaks, where the earlier peak consistently had higher amplitudes in TS (Shephard, 2013, pp. 102–103). With respect to children with ADHD who showed the smallest amplitude across outcomes here, it is interesting to note that the data presented by a prior study had the same pattern for this component at the central site around 300ms after flanker onset, as well as across flanker conditions (see figure 2 and 4 at Cz in Albrecht et al., 2008). Interestingly, in this dataset, the P3 component seemed reduced in children with ADHD (Albrecht et al., 2008, personal communication).

While there are some notable exceptions (Albrecht et al., 2008), many studies using simple choice response tasks in children do not find specific differences in N2 between ADHD and controls (e.g., Banaschewski et al., 2004; Broyd et al., 2005; Wiersema et al., 2006; Spronk et al., 2008). In our data, we saw a small frontal N2 component (not shown), but we did not find any clear negative modulation for incompatible and erroneous trials, or any group differences in the location and latency range of N2 that is typically present in flanker tasks in healthy young adults (e.g., Eichele et al., 2010b). Similarly, in this data we did not see a distinct ERN, or specific group differences therein, which may be explained by the clear developmental effect in this component, in the sense that our sample on average has an immature response (Davies et al., 2004). Due to the close interrelation between the ERN and the midfrontal N2, we can also speculate that frontal lobe maturation might affect N2 in the same way (Brydges et al., 2012; Tamnes et al., 2013).

We did separate analyses of the P3 subcomponents here to disentangle processing related to an early P3 component, representing more likely the orienting of attention to stimuli (Polich, 2007), and the later P3b/LPC reflecting response selection and other response-related processing (Falkenstein et al., 1994). The separation of stimulus- and response-locked LPC allows to study response selection/ orienting and response preparation separately (Verleger et al., 2005), which gives further insight into motor control in children with TS. It is possible that the greater increase in P3/LPC amplitude in the TS group reflects a stronger consolidation of the Flanker task in children with TS than in children with ADHD and control children (Johnson, 1984) and may suggest that children with TS employ greater resources in this process to maintain performance.

Individuals with TS frequently need to suppress emerging tics to achieve adequate psychosocial function. Other research has shown that children with TS have a generalized increase in cognitive control over motor activity (Mueller et al., 2006; Jackson et al., 2007) and enhanced control over their manual responses on a task-switching paradigm (Jackson et al., 2011), probably as a consequence of tic suppression. Here, we show that these adaptive effects already may happen earlier during stimulus evaluation, where an adaptation of the attentional system may result in higher attentional levels toward salient stimuli and an increased ability to suppress distracting information. This would in turn improve response selection.

The earlier discordant findings in the few previous studies of P3 in TS children may relate to different methods of recruiting the subjects (comorbidities, medication), and to differences in task selection (passive, active, visual, auditory, response mode; Luck, 2005), as well as different EEG/ERP post-processing and analysis. Here, use of ICA for artifact correction, and region of interest averaging allows for a clearer representation of a small, but robust ERP difference that is appreciable already in earlier work (Albrecht et al., 2008; Shephard et al., 2015).

We found smallest P3/LPC component amplitudes in the ADHD group, albeit not reaching significant difference levels compared with controls. This appears generally consistent with existing literature from several choice response tasks in this age group, including the Flanker task (Johnstone et al., 2010; Kratz et al., 2011). A reduced P3 in ADHD is considered reflective of diminished evaluative and processing capabilities (Brandeis et al., 2002; Lawrence et al., 2005; Johnstone et al., 2010; Kratz et al., 2011). Results from this group are heterogeneous however, for example a recent study using a Flanker task did not find differences on ERP or behavioral measures in children with ADHD compared to a control group (Johnstone and Galletta, 2013). Some inconsistencies may be related to study design, i.e., use of different compositions of clinical samples regarding age-range, sample size, medication status/type, gender distribution or comorbid disorders (Johnstone et al., 2013). However, amplitudes of children with ADHD become more like those of controls when motivated to perform well (Groom et al., 2010) and might have resulted in typical amplitude findings in our study.

We did not find that children with TS used a different strategy in prioritizing either speed or accuracy in compatible or incompatible trials and with respect to symptoms measured with the YGTSS, nor could we find significant correlations for speed or accuracy.

### Strengths and limitations of the study

All children were medication-naïve. Age and FSIQ differences did not readily explain group differences because groups were matched for age, as well as age and FSIQ were also used as covariates. The inclusion of children with ADHD is a strength of the study, because it allowed to illustrate the specificity of a higher P3 in children with TS, with and without comorbidity.

A limitation here is the relatively small sample size given the incidence, which led us to group TS+ADHD and TS only together. Ideally, the impact of comorbid conditions should be assessed separately, and in more detail, requiring larger sample sizes in future studies, probably best achievable through collaborative multi-site consortia. However, the fact that we did not find any significant differences between these subsamples in the dependent measures reported here justified the inclusion of children with TS only and those with additional ADHD in the same group. The relative lack of negative impact of comorbid ADHD on TS in our sample seems at variance with previous work reporting impaired ERPs (Shephard et al., 2015) and behavior (Roessner et al., 2007; Sukhodolsky et al., 2010; Greimel et al., 2011; Shephard et al., 2015) in participants with TS and ADHD. However, differences in mean age and gender distribution of the samples, as well as use of medication are different. Differences in task design and time on task may also play a role.

Many executive tasks are influenced by global changes in response caution, and motivation and error rates might fluctuate. The skills implemented to solve cognitive challenges may differ considerably in typically developing children from children with ADHD or TS. However, we tried to minimize these influences by keeping the time-on-trial to a minimum, and providing individual feedback after slow and after erroneous trials, respectively. During the experiment and upon debriefing there was no reason to suspect differences in motivation, attention or fatigue across groups and order of tasks was counterbalanced. Also, we used a robust estimate of PES (Dutilh et al., 2012), that discounts slow drifts.

## Conclusion

These findings provide further evidence that TS is not associated with widespread executive impairments, but presents robust evidence that adaptive changes, such as a heightened attentional capacity, are a core component of the TS disorder. In particular, we report a differential modulation of a P3-subcomponent that has not received much attention so far.

## Author contributions

Conception and design: HE, TE, IB, LS, KH, KP. Acquisition of data: HE, TE, MH, LS, HW, MW, KP. Analysis and interpretation: HE, TE, KP. Writing of article: HE, TE, KP. Critical review of article: HE, TE, IB, MH, LS, HW, MW, KH, KP. Final approval for publication: HE, TE, IB, MH, LS, HW, MW, KH, KP. Agreement to be accountable for all aspects of the work: HE, TE, IB, MH, LS, HW, MW, KH, KP.

### Conflict of interest statement

The authors declare that the research was conducted in the absence of any commercial or financial relationships that could be construed as a potential conflict of interest.
